# Associations between hydration status, body composition, sociodemographic and lifestyle factors in the general population: a cross-sectional study

**DOI:** 10.1186/s12889-022-13280-z

**Published:** 2022-05-05

**Authors:** Turgut Ekingen, Cynthia Sob, Christina Hartmann, Frank J. Rühli, Katarina L. Matthes, Kaspar Staub, Nicole Bender

**Affiliations:** 1 Institute of Evolutionary Medicine, University of Zurich, Winterthurerstrasse 190, 8057 Zurich, Switzerland; 2 Spital Bülach, Spitalstrasse 24, 8180 Bülach, Switzerland; 3 Institute for Environmental Decisions, ETH Zurich, Universitätsstrasse 22, 8092 Zurich, Switzerland; 4 Zurich Center for Integrative Human Physiology (ZIHP), University of Zurich, Winterthurerstrasse 190, 8057 Zurich, Switzerland

**Keywords:** Body hydration, Overweight, BIA, Sex differences, Physical activity

## Abstract

**Background:**

Whole-body hydration status is associated with several health outcomes, such as dehydration, edema and hypertension, but little is known about the nonclinical determinants. Therefore, we studied the associations of sex, age, body composition, nutrition, and physical activity on several body hydration measures.

**Methods:**

We assessed sociodemographic variables, dietary habits, and physical activity by questionnaire and body composition by bioelectric impedance analysis (BIA). We compared determinants between the sexes and calculated associations between determinants and BIVA hydration measures by multivariable linear regressions.

**Results:**

A total of 242 adults from the general population (age 18–94, 47% women) were included. Women were younger, smaller, lighter, and had a smaller BMI (kg/m^2^) than men (*p* < 0.05). Women had less muscle mass, less visceral fat mass and less extracellular and intracellular water than men (*p* < 0.001). Women showed less intracellular water per extracellular water than men, while men showed higher phase angle values than women (both *p* < 0.001). Men had a stronger association of hydration measures with physical activity than women. Both sexes showed a decrease in hydration measures with age.

**Conclusions:**

Sex, age, body composition, and physical activity influence body hydration. There seem to be differences in body water regulation between the sexes. Especially interesting are factors susceptible to preventive measures such as physical activity.

## Background

Sex- and age-dependent differences in body composition and hydration status have been described in the literature, but little is known about the interactions of these determinants and their variance [1–5]. In fact, whole-body hydration status plays a relevant role in health and disease [6]. For instance, it could be shown that high intracellular water content is associated with better functional performance and a lower frailty risk in elderly people [7]. In this age group even slight shifts or any imbalance of the hydration status can have health effects on the point of life-threatening consequences. Dehydration, for instance, can strongly impair cognitive functions and physical abilities of the body, and it has been associated with obesity, chronic diseases and decreased longevity in population-based studies [8].

In contrast, high extracellular water content due to environmental or behavioral factors can lead to severe complications in patients in the form of edema. Overhydration can cause severe organic and metabolic failures, which can be challenging to treat, even in hospitalized and monitored patients. Overhydration is especially relevant for patients with renal or cardiac diseases, but in population-based studies, overhydration was also associated with adverse outcomes such as hypertension [9]. Therefore, fluid management of the patient is of diagnostic relevance and therapeutic importance [10, 11].

The clinical detection of symptoms for dehydration has been utilized for years as a diagnostic tool. Furthermore, laboratory tests, such as blood or urine analyses, have been developed to diagnose and monitor the severity of body dehydration. However, no single symptom or test has proven sufficient to adequately diagnose dehydration in elderly patients, so the combination of several symptoms and tests is regarded as the most adequate procedure to diagnose dehydration [10, 12].

To evaluate body fluid volumes and hydration parameters in the general population and in patients bioelectrical impedance analysis (BIA) and bioelectrical impedance vector analysis (BIVA) are increasingly used in the last years [13, 14]. BIA measures the impedance (Z) of an alternating current flowing through the body and its hindrance by the soft tissue measured with four or eight electrodes placed on hands and feet in different ways, depending on the device (hand-to-hand, foot-to-foot, foot-to-hand, direct segmental). The impedance is divided into resistance (R) and reactance (Xc). R is related to total body water (TBW), while Xc is related to membrane integrity and tissue interfaces and therefore to tissue mass. The phase angle (PA) is the arc tan of Xc/R and represents a combination of body water distribution and soft tissue mass and is therefore a measure for tissue health. In BIA body composition measures such as fat mass (FM), fat free mas (FFM), skeletal muscle mass (SMM), TBW, and extracellular water (ECW) are calculated using population specific equations. The dependency of the underlying equations on specific populations, the differences in the measurement techniques (four points vs eight points, segmental, etc.), and the different sensitivities of the different devices makes it however difficult to directly compare data from one device to the other [15, 16].

In BIVA R and Xc of probands are plotted in confidence graphs of a comparative population. The Z vector represents PA in the graph and its length is inversely related to TBW or FFM. Such graphs allow to evaluate simultaneously hydration state and cell mass state of individuals or populations [17]. Contrary to BIA, BIVA is not dependent on population specific equations to calculate the outcomes.

Both techniques showed strengths and weaknesses in different populations such as children [4], athletes [14, 18–20], different ethnic groups [5], and patients [13, 21]. With the development of athlete-specific BIA-equations and BIVA-tolerance ellipses, for instance, both techniques showed helpful in monitoring training progress, nutritional status, fluid volumes, and hydration status in athletes [14]. Furthermore, it could be shown that patients with chronic kidney disease had a shorter and steeper mean vector in the BIVA tolerance graph compared to healthy probands [22]. PA seems to be a promising prognostic tool regarding malnutrition in cancer patients and critically ill patients [23].

The advantage of BIA and BIVA techniques is that they represent safe, rapid and non-invasive methods that help not only in diagnostics of body composition and hydration related disorders, but that also could lead to a better understanding of disease onset and progression [21]. Furthermore, a wider knowledge of these processes might lead to improvements in disease prevention or enable a more specific treatment of hydration-related pathologies [13].

What is less well understood to date are additional and potentially influencing factors on bioelectrical measurements such as diet or physical activity, especially if assessed by sex and in different age groups. In fact, lifestyle factors are known to influence body composition measures and disease risk [24]. The interplay of lifestyle factors with bioelectrical values could possibly have more public health relevance than is known to date, as preventive measures could potentially be derived from such results. Therefore, the overall objective of our study was to enhance our knowledge about the relative influence of life style determinants on body fluid volumes, hydration status, and the interplay between these factors in people from the general population of both sexes and of different age groups.

## Methods

The participants were recruited from an ongoing national nutritional study (Swiss Food Panel 2.0 [25]) by written invitation, by mailing lists from science communication events, and by media advertisement from the general population in the greater area of Zurich, Switzerland, in 2019 before the outbreak of COVID-19. The inclusion criteria were a minimal age of 18 years and a fluent command of the German language in reading and speaking skills. The participants were informed about the study procedures in written and oral form, and written informed consent was obtained prior to the data collection. The participants completed a food frequency questionnaire that also contained questions about the last time of drinking, physical activity at work and during free time [26], and sociodemographic variables such as sex, age and education level. The food frequency questionnaire derived from a previously published version [27, 28] that was based on the validated food frequency questionnaire of the Nurses’ Health Study [29]. The portion sizes were adapted according to the official Swiss recommendations [30]. The questionnaire asked about the mean food intake of the past year.

Body composition was analyzed with a segmental medical 8-point body composition analyzer (BIA) (Seca mBCA 515, Seca AG, Reinach, Switzerland) [31]. The participants stood barefoot on the four foot electrodes and placed both hands on the four hand electrodes of the device for the measurements.

The participants’ weight was measured with a balance incorporated in the BIA device, and body height was assessed with a standard stadiometer (Seca 274). Waist circumference (WC) was measured manually according to the WHO procedure [32]. The WC measurements were taken by trained research personnel at the midpoint between the lowest point of the ribcage and the highest point of the pelvis. A stretch-resistant hand-held tape with automatic retraction (Seca 201) was used. R and Xc values were obtained at 50 kHz for different body parts (right arm, left arm, trunk, right leg, left leg, right and left body side). These values were used in the prediction equations to calculate FFM, visceral fat mass (VFM), SMM, TBW, and ECW [15, 16, 33]. The prediction equations were developed using a four-compartment model, and with DXA, densitometry and dilution techniques as reference. The prediction equations were validated in a multi-ethnic population [16].

The measurements were performed at different times of the day, and the time was recorded. Participants were instructed not to eat or drink directly before the measurements and not to perform physical activity. The last time of eating, drinking, and of physical activity before the measurements was recorded. The study was approved by the Ethics Committee of ETH Zurich (EK 2019-N-08).

### Variables

The food frequency questionnaire was previously developed and implemented within the research frame of the Swiss Food Panel study [28]. From the food frequency questionnaire, we calculated a diet quality index from five food categories: fruits, vegetables, wholegrain products, meat, and sweet/salty snacks. For each category, the officially recommended minimum or maximum amount of weekly intake was used as the cutoff value, and a point was assigned if the recommendation was met. A score from 0 to 5 was built to reflect the overall healthiness of the diet [28]. For subgroup size reasons, the score was divided into three categories: unhealthy eating pattern (0–1), medium eating pattern (2–3), and healthy eating pattern (4–5). Education was assessed in the categories 1. mandatory education, 2. basic education, 3. professional training, 4. high school, 5. higher professional studies, 6. higher education, and 7. university. For subgroup size reasons, the data were dichotomized into primary/secondary education (1–4) and tertiary education (5–7). Physical activity in free time was assessed as 1. very light (almost no physical activity), 2. Light (e.g. walking, slow biking), 3. Moderate (e.g. running, biking), 4. Heavy (e.g. intensive running, intensive biking), and 5. very heavy (exhaustive activity several times per week) [26]. For subgroup size reasons, the data were divided into three categories: light (1–2), moderate (3), and heavy (4–5). The last time of drinking any kind of liquid was assessed as 1. within last hour, 2. 1 h ago, 3. 2 h ago, and 4. 3 or more hours ago.

Age was categorized in four quartiles. Body mass index (BMI) was calculated from weight and height as kg/m^2^ and classified according to the official WHO classification into underweight (BMI < 18.5 kg/m^2^), normal weight (BMI 18.5—< 25 kg/m^2^), pre-obesity (BMI 25—< 30 kg/m^2^) and obesity (BMI ≥ 30 kg/m^2^) [34]. Skeletal muscle mass index (SMI) was calculated as SMM divided by the square of height (kg/m^2^), fat free mass index (FFMI) was calculated as FMM divided by the square of height (kg/m^2^), and fat mass index (FMI) was calculated as FM divided by the square of height (kg/m^2^).

From the body fluid volumes and hydration parameters, ratios were calculated for further analyses, namely TBW/weight (Body mass, BM), TBW/FFM, ECW/TBW, intracellular water (ICW)/TBW, ECW/FFM, ICW/FFM, and ECW/ICW, R/height (H), and Xc/H.

### Statistics

To compare the variables between the two sexes, we used the chi-square test for categorical data (age quartiles), Kruskal–Wallis equality-of-populations rank test to assess differences between ordinal categorical variables (diet quality index, BMI categories, physical activity at work and in free-time, education, last time of drinking), a one-sample binomial test to assess the difference between the sample sizes of women and men, and Wilcoxon-Mann–Whitney tests between two means of not normally distributed variables (age, sitting time per day, BM, FMI, VFI, R, R/H, Xc, Xc/H, and the body fluid volume and hydration measure ratios described above). Normally distributed variables were compared with a two-sample t-test.

As several variables did not show a normal distribution we analyzed the correlations between body composition and bioelectrical impedance variables with Spearman’s rank correlations, using Bonferroni corrections of the p-values for multiple testing, separately for men and women.

For BIVA graphs, mean impedance vectors with 95% confidence ellipses were drawn from R/H and Xc/H for men and women separately [17]. We did not include the data of standard populations, as these were measured with other devices than ours. Differences between the sexes were analyzed by a two-sample Hotelling T-test [35]. Tolerance ellipses were drawn for men and women separately and by age quartiles, showing all individuals in the 50%, 75%, and 95% confidence intervals of a standard population. R/H and Xc/H were Z-transformed in order to allow a comparison with the data of the standard population. The standard population consisted of healthy middle aged and elderly Italians, and were therefore comparable to our sample [35].

We used multivariable linear regression models separately by sex to assess the association between each of the bioelectrical impedance measures (R, R/H, Xc, Xc/H, and PA), the body composition variables (BMI, SMI and FMI), and the life style variables diet quality index and physical activity level at free-time, as well as potential confounders (age quartiles, education level). Not normally distributed variables were square-root or ln transformed before including them in the models (R, R/H, Xc, Xc/H, BMI, SMI, and FMI).

## Results

In total, *n* = 242 participants from the general population (47% females) were included in the study. Sociodemographic characteristics and variable descriptions by sex are given in Tables 1 and 2. Women were slightly younger than men (mean (SD) 50.2 y (19.7) vs 57.9 y (18.2), *p* < 0.01), and there was no difference in educational level or in physical activity at work or in sitting time per day (*p* > 0.05) (Table 1). Men showed more physical activity in free time, and women showed a healthier eating pattern (*p* < 0.05). Men were heavier (mean (SD) 79.6 kg (11.5) vs 62.9 kg (8.7), *p* < 0.001), taller (mean (SD) 1.77 m (0.07) vs 1.65 m (0.06), p < 0.001) and had a higher BMI (*p* < 0.001) than women. In men, 48.8% had a BMI ≥ 25 units, while in women, this value was 20.3% (Table 2).

**Table 1:** Sociodemographic and life style variables. In bold are significant results (*p* < 0.05)

Variable	Women Mean (SD)		Men Mean (SD)				Difference*
Sample size (n)	114		128				*p* > 0.05
Age (range)	18–87		20–94				
Age (mean ± SD)	50.2 (19.7)		57.9 (18.2)				***p* < 0.01**
Age categories (quartiles)	Freq	Percent	Freq	Percent			***p* < 0.001**
First quartile	39	34.2	23	18.0			
Second quartile	31	27.2	33	25.8			
Third quartile	22	19.3	35	27.3			
Fourth quartile	22	19.3	37	28.9			
Education	Freq	Percent	Freq	Percent			*p* > 0.05
Primary and secondary education	39	37.1	41	33.9			
Tertiary education	66	62.9	80	66.1			
Physical activity at work	Freq	Percent	Freq	Percent			*p* > 0.05
Light	43	39.5	62	49.6			
Moderate	35	32.1	39	31.2			
Heavy	31	28.4	24	19.2			
Physical activity in free time	Freq	Percent	Freq		Percent		***p* < 0.05**
Light	14	12.5	18		14.2		
Moderate	62	55.4	43		33.9		
Heavy	36	32.1	66		52.0		
Sitting time per day (hours ± SD)	7.6 (3.3)		7.5 (3.4)				*p* > 0.05
Healthy eating index	Freq	Percent	Freq		Percent		***p* < 0.001**
Unhealthy	44	39.6	76		60.8		
Medium	49	44.1	43		34.4		
Healthy	18	16.2	6		4.8		
Last time drinking	Freq	Percent	Freq. Percent				*p* > 0.05
Recently	51	45.1	35			27.6	
Medium	21	18.6	43			33.9	
Long ago	41	36.3	49			38.6	

^*^We used a one-sample binomial test to assess the difference between sample sizes, Wilcoxon-Mann–Whitney test to assess differences in means, and the Kruskal–Wallis equality-of-populations rank test to assess differences between categorical variables

**Table 2:** Anthropometry and body composition. In bold are significant results (*p* < 0.05)

**Variable**	**Women** **Mean (SD)**		**Men** **Mean (SD)**		*Difference
Height (m)	1.65 (0.06)		1.77 (0.07)		***p* < 0.001**
BM (kg)	62.9 (8.7)		79.6 (11.5)		***p* < 0.001**
BMI (kg/m^2^)	Freq	Percent	Freq	Percent	***P* < 0.001**
< 18.5	5	4.4	1	0.8	
18.5—< 25	85	75.2	62	50.4	
25—< 30	18	15.9	47	38.2	
≥ 30	5	4.4	13	10.6	
SMI (kg/m^2^)	6.93 (0.8)		9.23 (1.2)		***p* < 0.001**
FFMI (kg/m^2^)	15.6 (1.3)		19.4 (1.9)		***p* < 0.001**
FMI (kg/m^2^)	7.4 (2.6)		6.1 (2.5)		***p* < 0.001**
VFM (l)	0.97 (0.7)		2.5 (1.6)		***p* < 0.001**
TBW/BM	0.51 (0.05)		0.56 (0.05)		***p* < 0.001**
TBW/FFM	0.75 (0.01)		0.74 (0.01)		***p* < 0.001**
ECW/TBW	0.46 (0.03)		0.43 (0.02)		***p* < 0.001**
ICW/TBW	0.54 (0.03)		0.57 (0.02)		***p* < 0.001**
ECW/ICW	0.85 (0.09)		0.76 (0.08)		***p* < 0.001**
ECW/FFM	0.34 (0.02)		0.32 (0.02)		***p* < 0.001**
ICW/FFM	0.41 (0.02)		0.42 (0.02)		***p* < 0.001**
R (Ω)	661.4 (57.6)		532.7 (56.8)		***p* < 0.001**
R/H (Ω/m)	400.8 (36.3)		301.4 (32.6)		***p* < 0.001**
Xc (Ω)	56.7 (7.9)		50.3 (9.0)		***p* < 0.001**
Xc/H (Ω/m)	34.3 (4.8)		28.5 (5.2)		***p* < 0.001**
PA (°)	4.9 (0.6)		5.4 (0.9)		***p* < 0.001**

^*^We used the Kruskal–Wallis equality-of-populations rank test to assess differences between BMI categories, two sample t-tests to assess differences between heights and phase angles, and the Wilcoxon-Mann–Whitney test to assess differences between the means of the other variables

**Fig. 1:**
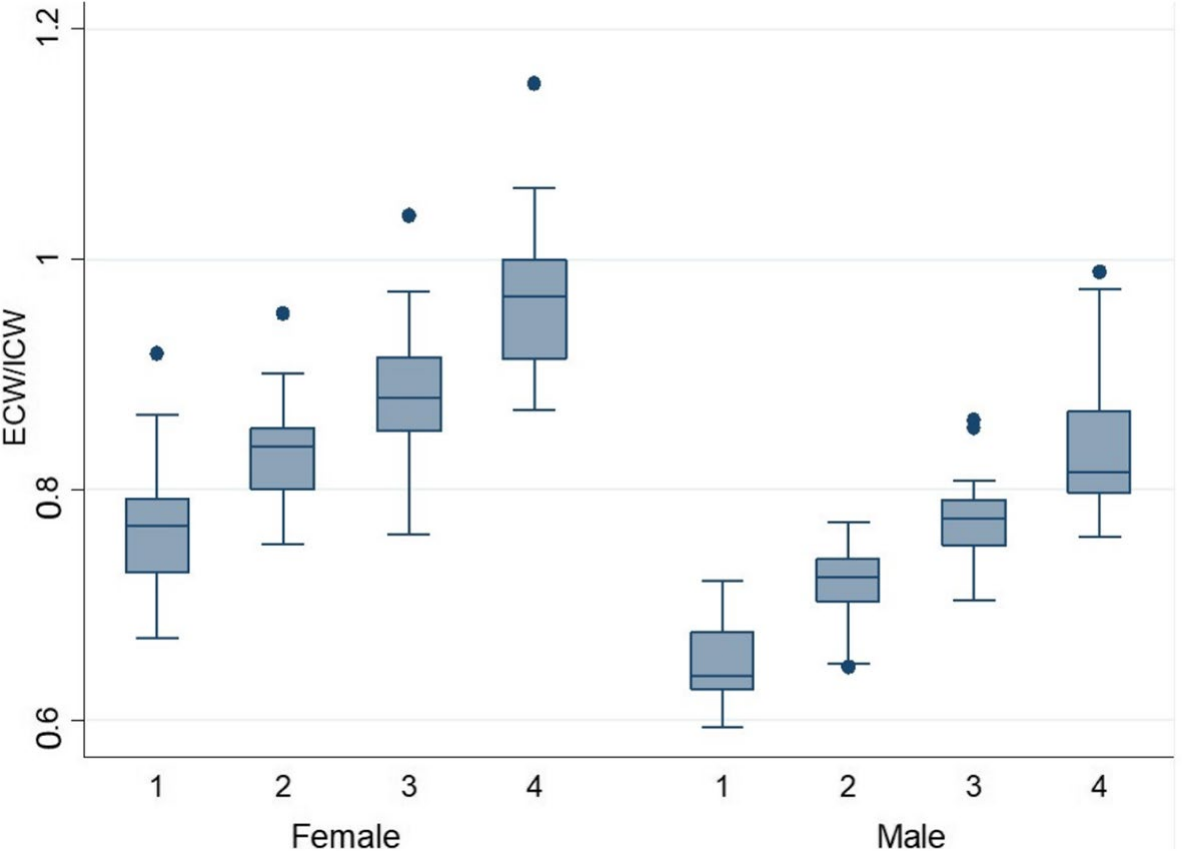
Boxplots of extracellular water per fat free mass (ICW/FFM), per age quartile in women and men. 1 = youngest quartile, 4 = oldest quartile

In terms of body composition, men had a higher SMI (mean (SD) 9.23 kg/m^2^ (1.2) vs 6.93 kg/m^2^ (0.8), *p* < 0.001, FFMI (mean (SD)) 19.4 kg/m^2^ (1.9) vs 15.6 (1.3), p < 0.001 VFM (mean (SD)) 2.5 l (1.6) vs 0.97 l (0.7), *p* < 0.001), and a lower FMI (mean (SD) 6.1 kg/m^2^ (2.5) vs 7.4 (2.6), p < 0.001), than women. TBW/weight was higher in men, while TBW/FFM was higher in women, meaning that the total water volume of the body was higher in women if fat mass was not considered. ECW/TBW was higher in women than in men, while ICW/TBW was higher in men than in women (*p* < 0.001). Likewise, ECW/FFM was higher in women than in men, while ICW/FFM was higher in men than in women (*p* < 0.001). ECW/ICW was higher in women than in men (*p *< 0.001) (Table 2) and increased with age in both sexes (Fig. 1).

**Fig. 2:**
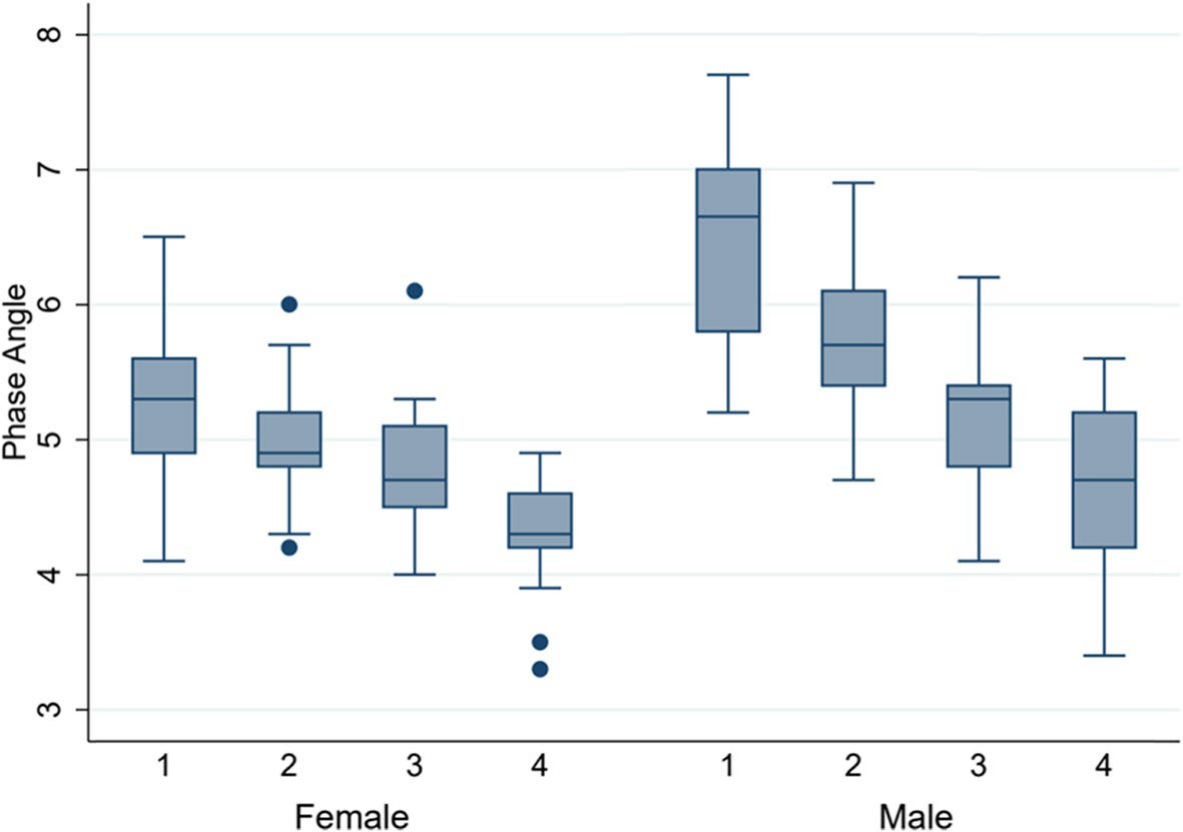
Boxplots of phase angle per age quartile in women and men. 1 = youngest quartile, 4 = oldest quartile

**Fig. 3:**
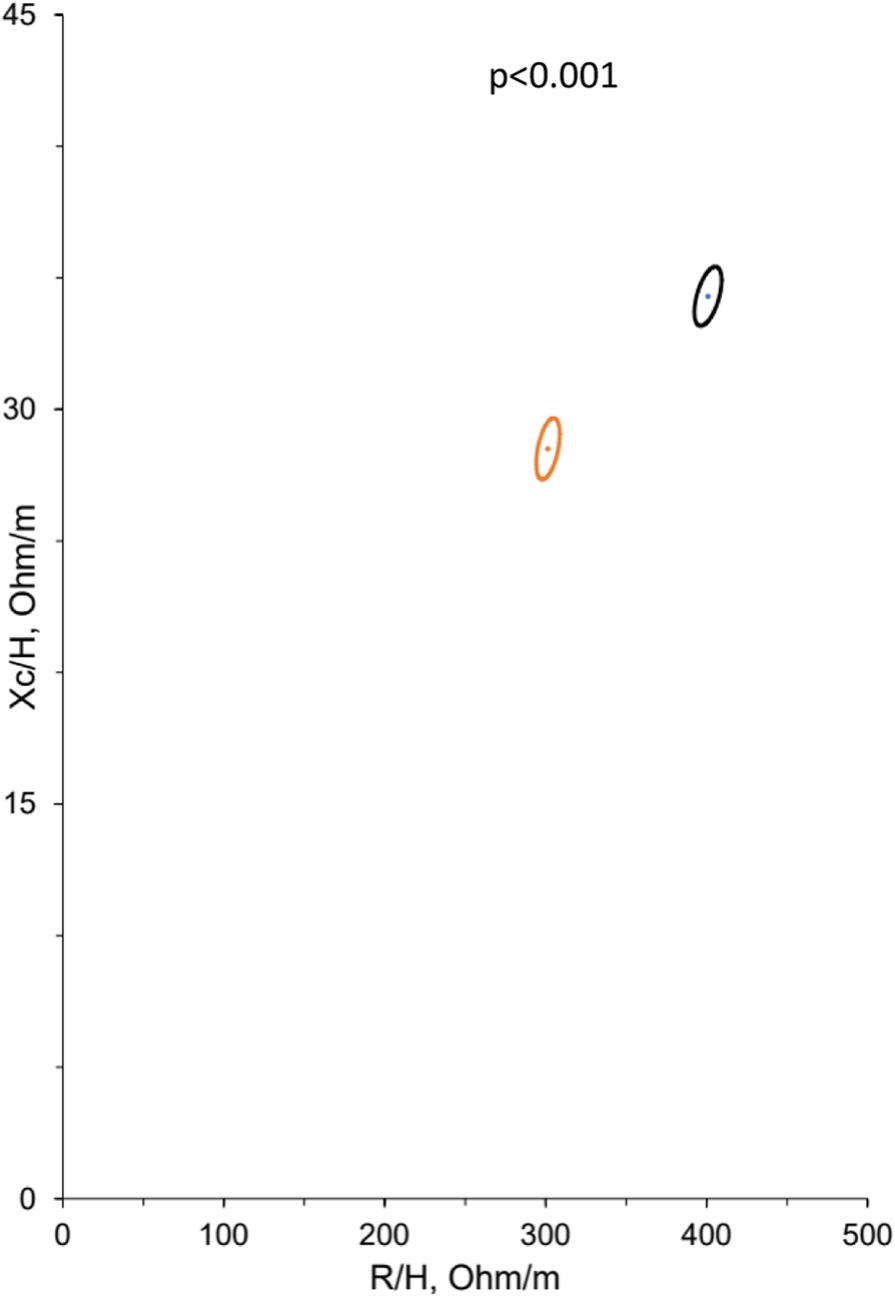
The 95% confidence ellipses for the mean impedance vectors of men (red) in comparison with women (black). R/H: height-adjusted resistance; Xc/H: height-adjusted reactance. The *p*-value is from the two-samples Hotelling’s T^2^-Test

**Fig. 4:**
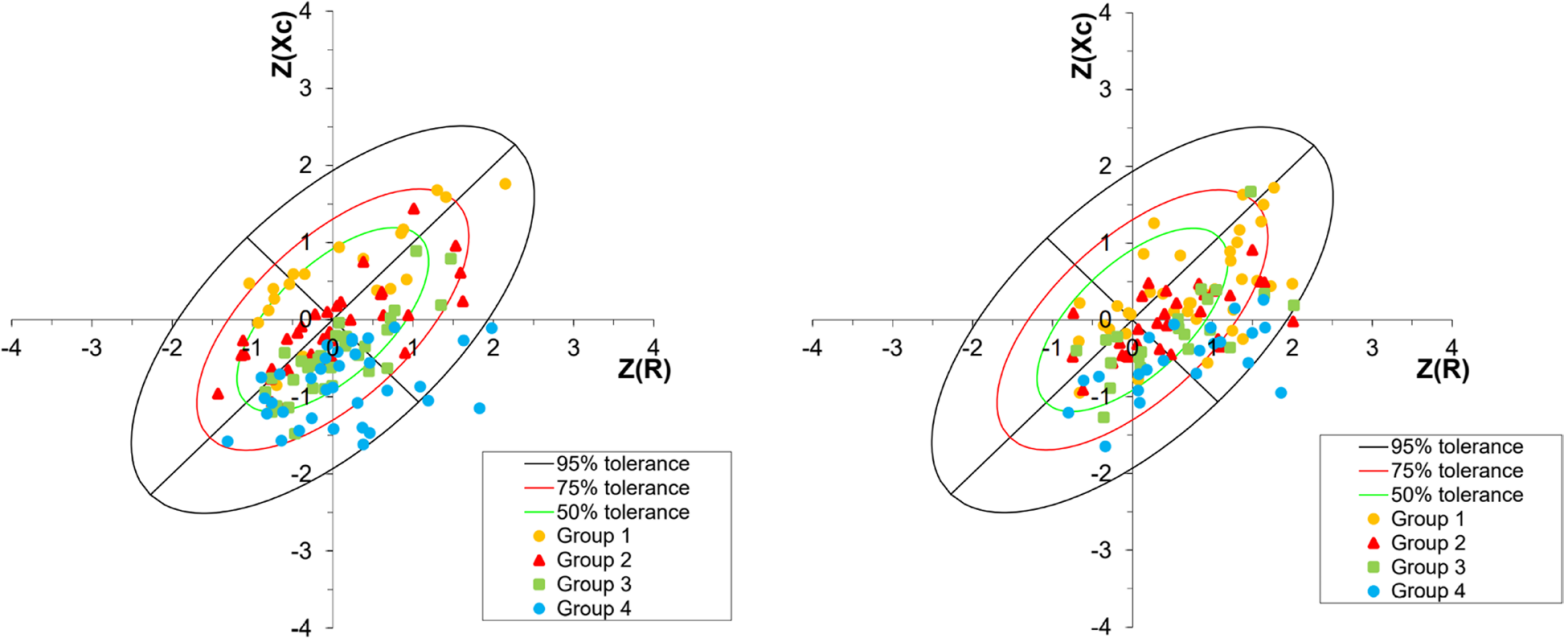
Scatterplot of individuals in four age quartiles of men (left) and women (right), plotted on the 50%, 75%, and 95% tolerance ellipses of the corresponding reference populations. Group 1 (yellow): first quartile (youngest); group 2 (red): second quartile; group 3 (green): third quartile; group 4 (blue): fourth quartile (oldest). Z(R): Z-transformed resistance; Z(Xc): Z-transformed reactance

Bioelectrical measures (R, R/H, Xc, and Xc/H) were all significantly higher in women than in men. Contrary to this, PA (mean (SD)) was higher in men (5.4° (0.9)) than in women (4.9° (0.6)) (Table 2) and decreased in both sexes over age (Fig. 2). The 95% confidence ellipses for the mean impedance vectors showed a significant difference between men and women (*p* < 0.001) (Fig. 3). The scatterplot of individuals plotted on the 50%, 75%, and 95% tolerance ellipses of the Italian reference population divided by sex and age groups showed that there is an age gradient along the Z(R) axis, but not along the Z(Xc) axis. Older individuals show less body cell mass, but not less hydration compared to the reference population, while younger individuals are well within the 75% percentile. This result is especially pronounced in men (Fig. 4).

The Spearman correlations between body composition and bioelectrical variables showed differences between the sexes. In men, R and R/H correlated with all outcomes apart of FMI, while Xc and Xc/H correlated only with ECW. In women, Xc and Xc/H were negatively correlated to BMI and FMI. While in men no bioelectrical value correlated with FMI, in women apart from Xc and Xc/h also PA correlated to FMI. PA positively correlated in both sexes with FFMI, SMI and ICW (Table 3).

**Table 3:** Spearman correlations between body composition and bioelectrical variables

**Men**								
	**Weight**	**BMI**	**FFMI**	**SMI**	**FMI**	**TBW**	**ECW**	**ICW**
**R**	-0.502***	-0.633***	-0.837***	-0.729***	-0.216	-0.596***	-0.670***	-0.475***
**R/H**	-0.640***	-0.555***	-0.782***	-0.728***	-0.152	-0.800***	-0.875***	-0.667***
**Xc**	-0.122	-0.166	0.036	0.190	-0.231	0.046	-0.394***	0284
**Xc/H**	-0.215	-0.106	0.064	0.180	-0.177	-0.092	-0.520***	0.145
**PA**	0.157	0.212	0.535***	0.643***	-0.110	0.374**	-0.044	0.567***
**Women**								
	**Weight**	**BMI**	**FFMI**	**SMI**	**FMI**	**TBW**	**ECW**	**ICW**
**R**	-0.562***	-0.629***	-0.664***	-0.556***	-0.341*	-0.512***	-0.685***	-0.331*
**R/H**	-0.678***	-0.505***	-0.645***	-0.603***	-0.208	-0.739***	-0.863***	-0.557***
**Xc**	-0.301	-0.430***	0.093	0.252	-0.595***	0.076	-0.387**	0.356**
**Xc/H**	-0.407***	-0.359**	0.064	0.180	-0.498***	-0.113	-0.543***	0.168
**PA**	0.023	-0.047	0.607***	0.726***	-0.447***	0.448***	0.024	0.654***

*BMI* Body mass index, *FFMI* Fat free mass index, *SMI* Skeletal muscle mass index, *FMI* Fat mass index, *TBW* Total body water, *ECW* Extracellular water, *ICW* Intracellular water, *R* Resistance, *Xc* Reactance, *H* Height, *PA* Phase angle

^*^p < 0.05, **p < 0.01, ***p < 0.001. All *p*-values are Bonferroni-corrected for multiple testing

The multiple regression showed no associations between R/H, lifestyle factors, and potential confounders in both sexes, apart for the oldest age group compared to the youngest (*p* < 0.05), indicating a loss of hydration in the oldest age group compared to the youngest in both sexes. This result confirms the visual impression of the tolerance ellipses in Fig. 4. The association with BMI went lost if corrected for all the other variable in the model (Table 4).

**Table 4:** Associations between bioelectrical variables, lifestyle factors, and potential confounders, by sex. Multiple regression coefficients with 95% confidence intervals (95% CI) and p-values. In bold are *p*-values under 0.05

Variable	Coefficient	95% CI	*p*-value	Coefficient	95% CI	*p*-value
**R/H**	**Women**			**Men**		
Diet quality index:						
Unhealthy, baseline						
Medium	0.03	-0.04, 0.09	> 0.05	0.02	-0.04, 0.07	> 0.05
Healthy	0.03	-0.05, 0.11	> 0.05	0.09	-0.03, 0.21	> 0.05
Physical activity:						
Low, baseline						
Medium	0.07	-0.02, 0.16	> 0.05	0.02	-0.07, 0.10	> 0.05
High	0.02	-0.08, 0.11	> 0.05	0.01	-0.07, 0.09	> 0.05
BMI	-0.16	-0.41, 0.09	> 0.05	-0.04	-0.25, 0.17	> 0.05
Age quartiles:						
Youngest, baseline						
Second	0.01	-0.07, 0.08	> 0.05	0.01	-0.07, 0.08	> 0.05
Third	0.07	-0.01, 0.15	> 0.05	0.02	-0.06, 0.10	> 0.05
Oldest	0.11	0.02, 0.20	** < 0.05**	0.09	0.01, 0.17	** < 0.05**
Education:						
Mandatory, baseline						
Higher	-0.02	-0.08, 0.04	> 0.05	0.02	-0.03, 0.08	> 0.05
**Xc/H**						
Diet quality index:						
Unhealthy, baseline						
Medium	-0.06	-1.89, 1.76	> 0.05	-0.01	-1.56, 1.53	> 0.05
Healthy	-1.21	-3.52, 1.11	> 0.05	2.11	-1.31, 5.53	> 0.05
Physical activity:						
Low, baseline						
Medium	1.28	-1.34, 3.89	> 0.05	1.78	-0.67, 4.23	> 0.05
High	1.70	-1.05, 4.44	> 0.05	2.91	0.54, 5.28	** < 0.05**
BMI	-8.49	-15.61, -1.38	** < 0.05**	-1.78	-7.87, 4.30	> 0.05
Age quartiles:						
Youngest, baseline						
Second	-2.41	-4.50, -0.32	** < 0.05**	-4.00	-6.20, -1.80	** < 0.05**
Third	-3.65	-6.00, -1.31	** < .0.05**	-6.18	-8.43, -3.94	** < 0.05**
Oldest	-6.10	-8.69, -3.51	** < 0.05**	-8.88	-11.07, -6.68	** < 0.05**
Education:						
Mandatory, baseline						
Higher	-1.22	-2.91, 0.47	> 0.05	0.39	-1.20, 1.96	> 0.05
**PA**						
Diet quality index:						
Unhealthy, baseline						
Medium	-0.12	-0.32, 0.08	> 0.05	-0.07	-0.24, 0.11	> 0.05
Healthy	-0.19	-0.44, 0.06	> 0.05	-0.03	-0.42, 0.37	> 0.05
Physical activity:						
Low, baseline						
Medium	0.17	-0.11, 0.46	> 0.05	0.50	0.22, 0.78	** < 0.05**
High	0.39	0.09, 0.69	** < 0.05**	0.84	0.57, 1.11	** < 0.05**
BMI	0.82	0.05, 1.60	** < 0.05**	2.32	1.61, 3.02	** < 0.05**
Age quartiles:						
Youngest, baseline						
Second	-0.32	-0.54, -0.09	** < 0.05**	-0.77	-1.03, -0.52	** < 0.05**
Third	-0.49	-0.74, -0.23	** < 0.05**	-1.06	-1.32, -0.08	** < 0.05**
Oldest	-1.01	-1.29, -0.73	** < 0.05**	-1.63	-1.88, -1.37	** < 0.05**
Education:						
Mandatory, baseline						
Higher	-0.18	-0.36, 0.00	> 0.05	-0.11	-0.29, 0.07	> 0.05

*R/H* Resistance per height, *Xc/H* Reactance per height, *PA* Phase Angle, *BMI* Body Mass Index

For Xc/H the model showed a positive association with physical activity in men (highest level against lowest level, *p* < 0.05), indicating more cell mass with high levels of physical activity. In women there was a negative association with BMI (*p* < 0.05). In both sexes there was a negative association with all age groups (*p* < 0.05), indicating less cell mass with increasing age (Table 4).

For PA there was a positive association with physical activity in both sexes (*p* < 0.05), indicating more tissue health with increasing physical activity. This result was true in women for the highest level compared to the lowest (*p* < 0.05), while in men also the moderate level was significant compared to the lowest (*p* < 0.05). In both sexes there was a positive association with BMI (*p* < 0.05) and a negative association with all age groups (*p* < 0.05), indicating less tissue health with increasing age (Table 4).

## Discussion

Our results show associations of bioelectrical hydration measures and body composition parameters with sex, age and physical activity in a sample of the general population in Switzerland. All body composition and hydration parameters differed significantly between the sexes. ECW/ICW is a measure of fluid volume shift between the compartments and can be an indicator for systemic inflammation, pathologies or aging [36]. In our study the proportion of ECW/ICW was higher in women than in men, and increased with age in both sexes. In contrast, phase angle, a measure for tissue health, was higher in men than in women, and decreased with age in both sexes. Similar results were found in larger studies of healthy subjects [37, 38].

The R/Xc scatterplots by sex and subdivided by age groups showed that the shift of the age groups along the Z/Xc axis was stronger than the shift along the Z/R axis, especially in men. This means that the decrease in cell mass with increasing age was stronger than the decrease in hydration with increasing age for both sexes, but more in men. This result could indicate a pattern of possible increasing sarcopenia with age, a common consequence of malnutrition in old age [23, 39]. The common pattern found for ECW/ICW and for phase angle, as well as for the scatterplots, seems therefore to be a combination of sex hormonal and aging effects. Sex hormones are responsible not only for the physiological hydration differences between the sexes but also for the differences in hydration status and fluid volumes between pre- and postmenopausal women [1, 40]. Concerning muscle mass, a part from sex hormones, also genetic factors might play a role in the difference between men and women [41]. In fact, the result that in men the decrease of PA and therefore muscle mass with increasing age was stronger than in women was also observed in a population of older adults [42].

In the multilinear models the association with physical activity was present for Xc/H in men, but not in women, and was stronger for PA in men than in women. In both models age was significantly associated for both sexes with the outcomes. These results indicate that there is an association between physical activity and tissue mass/health which is age dependent in both sexes, but which is stronger in men. A similar sex difference of the impact of physical activity on bioelectrical values which is only present or stronger in men than in women was also found in athletes [43, 44]. Such findings can inform sex specific preventive public health measures. For instance, a recent meta-analysis showed that men gained more absolute muscle mass and muscle strength than women after resistance training in older adults [45].

Interestingly, diet quality did not show any association with the bioelectric outcomes in our models, neither for men nor for women. We did not find comparable studies in the general population. Most studies assessing the impact of diet or supplements on body composition or hydration with BIVA were carried out in patients and found diverging results, from no difference to an improvement of body composition after intervention [46–50]. Also, in studies carried out in athletes, supplementation with proteins or other nutrients showed inconclusive results [51, 52]. More studies are needed to assess the impact of diet on bioelectrical measurements. As most BIVA outcomes show sex differences, these investigations should be carried out by sex.

Our study comes with strengths and limitations. To our knowledge, our study is the first to include different lifestyle factors, such as diet and physical activity, in an analysis of bioelectric hydration parameters and body fluid volumes in the general population. Other studies compared different hydration and fluid volume measurements and ratios with each other, showing that there are associations with sex, age, and body composition, but they were not assessed for lifestyle factors. Furthermore, most studies are carried out in specific populations like athletes, patients or children [2, 4, 53]. Another strength of our study is the use of an eight-point BIA device that delivers high quality measurements, and the wide range of age and BMI of our study population. As only people of European ancestry participated in our study, there is no risk of confounding by ethnicity.

A limitation of the present study is the limited sample size and the fact that the study consisted of an unweighted convenience sample from the general population. The sample is therefore not representative for Switzerland. Another limitation of the study is that there was no quantitative registration of fluid intake per day in the food frequency questionnaire, as fluid intake might influence the outcome of BIA measurements [3]. We considered the last point in time of fluid intake in our analysis to account for this potential influence. However, the last time of drinking did not show an influence on the results (data not shown). Subsequent studies should nevertheless take fluid intake into consideration, as this factor could deliver valuable information. We also did not have information on women’s hormonal status or on participants’ medical conditions or medication intake. These factors might influence body composition and hydration status of the participants [1, 8].

BIA is not considered the gold standard for body composition measurement, and for body hydration in particular, no gold standard exists to date [3, 14, 54–56]. Nevertheless, BIA and BIVA are increasingly assessed and used in studies on body hydration and body fluid volumes, showing strengths and limitations of the technique [3, 4, 13, 14, 20], and reference values from the general population are produced to compare to study results [5, 31]. Among the most often expressed concerns about the BIA technique is that the calculations of the body composition outcomes are dependent from population specific equations, and that different devices from different manufacturers might deliver different results. For instance, it was shown that BIA measurements from single-frequency devices and from multi-frequency devices carried out in the same populations were not comparable and should therefore not be used interchangeably [57]. It is therefore generally important not to directly compare results obtained with different devices and to respect the limitations of the method. Furthermore, there is a call for standardized comparative population data and for standardized measurement protocols for BIA and BIVA applications [13, 14, 21].

## Conclusions

We found sex specific differences in body composition, body fluid volumes, and hydration parameters, as well as associations with age and physical activity. Particularly, measures of cell mass were lower in higher age classes in men than in women, while physical activity showed a stronger association with measures of tissue health in men than in women. The role of lifestyle factors in body hydration over the life course and their sex specific metabolic pathways have to be investigated further. Such data might inform therapeutic and preventive interventions in the future, in line with precision medicine efforts, as well as public health measures.

## Data Availability

The datasets used and/or analysed during the current study are available from the corresponding author on reasonable request.

## Abbreviations

BIA: Bioelectrical Impedance Analysis
BIVA: Bioelectrical Impedance Vector Analysis
BMI: Body Mass Index
BM: Body Mass
BW: Body Weight
ECW: Extracellular Water
ETH: Eidgenössische Technische Hochschule (University for Science and Technology)
FFMI: Fat Free Mass Index
FM: Fat Mass
FMI: Fat Mass Index
H: Height
ICW: Intracellular Water
PA: Phase Angle
R: Resistance
SD: Standard Deviation
SMI: Skeletal Mass Index
SMM: Skeletal Muscular Mass
TBW: Total Body Water
VFI: Visceral Fat Index
VFM: Visceral Fat Mass
WC: Waist Circumference
Xc: Reactance
